# Effects of social intention on movement kinematics in cooperative actions

**DOI:** 10.3389/fnbot.2013.00014

**Published:** 2013-10-04

**Authors:** François Quesque, Daniel Lewkowicz, Yvonne N. Delevoye-Turrell, Yann Coello

**Affiliations:** ^1^Université Lille Nord de FranceLille, France; ^2^Unité de Recherche en Sciences Cognitives et Affectives, Université de Lille 3Villeneuve d’Ascq, France

**Keywords:** motor planning, social interaction, kinematics, reachable space, intention

## Abstract

Optimal control models of biological movements are used to account for those internal variables that constrain voluntary goal-directed actions. They, however, do not take into account external environmental constraints as those associated to social intention. We investigated here the effects of the social context on kinematic characteristics of sequential actions consisting in placing an object on an initial pad (preparatory action) before reaching and grasping as fast as possible the object to move it to another location (main action). Reach-to-grasp actions were performed either in an isolated condition or in the presence of a partner (*audience effect*), located in the near or far space (*effect of shared reachable space*), and who could intervene on the object in a systematic fashion (*effect of social intention effect*) or not (*effect of social uncertainty*). Results showed an absence of *audience effect* but nevertheless an influence of the social context both on the main and the preparatory actions. In particular, a “localized” *effect of shared reachable space* was observed on the main action, which was smoother when performed within the reachable space of the partner. Furthermore, a “global” effect of *social uncertainty* was observed on both actions with faster and jerkier movements. Finally, *social intention* affected the preparatory action with higher wrist displacements and slower movements when the object was placed for the partner rather than placed for self-use. Overall, these results demonstrate specific effects of action space, social uncertainty and social intention on the planning of reach-to-grasp actions, in particular on the preparatory action, which was performed with no specific execution constraint. These findings underline the importance of considering the social context in optimal models of action control for human–robot interactions, in particular when focusing on the implementation of motor parameters required to afford intuitive interactions.

## INTRODUCTION

It is five o’clock and a waiter is faced with the task of clearing a littered table, after a group of customers depart. Through experience, the waiter has learned to produce grip force levels that are adapted to the needs of commonly manipulated objects and to follow hand trajectories that are adapted to the cluttered environment. Empirical studies in laboratory settings have confirmed that physical parameters of an object such as size (Marteniuk et al., 1990; Chieffi and Gentilucci, 1993; Pryde et al., 1998; Armbrüster and Spijkers, 2006), weight (Eastough and Edwards, 2007), shape (Gentilucci et al., 1991), and even texture (Fikes et al., 1994) influence the dynamical aspects of motor performance, in particular the reach-to-grasp motor kinematics. Nevertheless, other internal variables have also been shown to modify motor planning of reaching actions such as the comfort of final posture (Rosenbaum et al., 1990) and the smoothness of movement trajectory (Flash and Hogan, 1985). Most importantly for the matter here, the intention that drives an action can also modulate motor kinematics (Becchio et al., 2008a). Indeed, our waiter may not grasp a glass in the same way if he has the intention to give it to a customer (in this case, the movement may be slow and accurate) or to grip it quickly to put it on a large shelf in order to clean the table before the arrival of the next set of customers. Hence, intention in action as described by Searle (1983) and Jeannerod (2006) represents one category of internal variables that may substantially influence the planning of voluntary action because it encapsulates the fundamental reason of acting.

It is the case that these internal parameters are poorly taken into account in the computational modeling of motor control. Indeed, optimal control models of biological movement are successful in predicting empirical findings such as movement adjustments to unexpected changes in object position or size, and/or responses to global perturbations (Shadmehr and Mussa-Ivaldi, 1994), and also in modeling the structure of motor variability in function of the physical properties of an object and/or its environment (Gordon et al., 1994; Messier and Kalaska, 1999; Van Beers et al., 2004) as well as the generic motor laws associated to a given situation (Lacquaniti et al., 1983). However, optimal control models are poorly adapted to predict the empirical data obtained in interactive situations (Friston, 2011), rendering human–robot interactions massively unidirectional (Chaminade and Cheng, 2009). Indeed, during social interaction, Boucher et al. (2012) showed that human agents placed in a cooperative context are sensitive to the predictive information provided by the direction of gaze of their partners, even when interacting with robots. Furthermore, motor intention influences movement kinematics in such a way that not only the goal of individual actions can be anticipated by a perceiver (Lewkowicz et al., 2013), but also coordinated actions involving several agents can be performed (Knoblich and Sebanz, 2008; Vesper et al., 2010). Thus, it seems important for artificial social intelligence to develop (1) our knowledge of the specific effects that motor intention has on movement kinematics during a true social interactive task and (2) to provide solid guidelines for the development of optimal control models that will be able to implement intention in action in those artificial agents that need to cooperate intuitively with biological organisms.

The effect of motor intention on arm kinematics is a phenomenon that was first reported by Marteniuk and colleagues in the late 1980s (Marteniuk et al., 1987). In this study, they showed that reach-to-grasp movements toward an object differed according to whether the grasped object was afterward thrown away into a large box or placed into a well. More specifically, results showed that the arm trajectories (i.e., the resultant velocity profile of the wrist) were modulated with an increase in duration of the main deceleration phase of the trajectory when task demands required greater precision. These results did not support a simple scaling procedure in the temporal domain as what would be expected with the optimal control models of biological movements. Rather, their results supported a view of movement production as relatively specific to the past experiences of the performer and the constraints of the future task. In the continuity of this pioneering study, other studies later reported that not only the final intention but also the characteristics of the second component of a sequential movement could lead to early variants in the first component of the sequence. The effects of a second movement on the first were described in non-manipulative tasks, i.e., pointing (Orliaguet et al., 1996) and writing (Orliaguet et al., 1997). This back propagation effect was also shown in grasping movements when participants were required to grasp (1) an object to eat it or move it (Naish et al., 2013), (2) an object to lift or insert it into a niche (Ansuini et al., 2006), or (3) a bottle with the intention to use it or to dispose from it (Ansuini et al., 2008; Schuboe et al., 2008). More recent studies have finally shown that the final purpose of a grasping action strongly influences the kinematics of both the transport phase and the characteristics of the hand shaping, i.e., the manipulation component (Ansuini et al., 2006). As a consequence, when observing an action performed by someone else, it seems possible from early kinematics to anticipate the goal of the action, i.e., much before the entire action is accomplished (Méary et al., 2005; Manera et al., 2011; Sartori et al., 2011; Lewkowicz et al., 2013).

Recently, Georgiou et al. (2007) showed that the social context while performing a voluntary motor action has also an effect on the kinematics of a reach-to-grasp component of a motor sequence. More specifically, they found that the kinematics of an identical motor action (reaching-to-grasp a wooden block) was different in a cooperative vs a competitive task, and both kinematics patterns could be distinguished from a similar action performed by the participants in isolation. In the same vein, an effect of social intention was reported for movement kinematics when comparing reach-to-grasp actions in a social (passing an object to another person) and a non-social context (putting an object in a concave base; Becchio et al., 2008a). Furthermore, social affordances can affect movement kinematics even when no social interaction is expected (Ferri et al., 2011). In fact, the mere presence of an active conspecific appears sufficient in certain cases to induce changes in movement kinematics (Gianelli et al., 2011). In particular, when participants were requested to grasp an object and then move it to a container, the presence of a person unexpectedly stretching out the arm – as for a social request – affected motor kinematics of those actions that were directed toward the object only (Sartori et al., 2009b). Interestingly, this pattern of results was not observed when humans interacted with robotic agents, a situation that influenced neither arm trajectories, nor kinematic profiles, suggesting a lack of true social interaction when humans interact with robotic systems. Considered together, these data support the view that specific kinematic patterns characterize and distinguish actions performed in a social and communicative context from those actions executed with a purely individual intent. One reason for this effect of social context on kinematics could be that communicative actions are intended to be identified by a partner and to engage him/her in a communication process (Sartori et al., 2009a). Accordingly, by simply observing the movements performed by others, one might be able to comprehend what they are planning to do and thus, know how one should act in response (Becchio et al., 2012). This point of view fits well with the observation that social effects on reach-to-grasp movement depend on the spatial location of the other person. In particular, latencies in responding have been shown to be significantly shorter when partners are in positions allowing them to easily reach for the object (Gianelli et al., 2013). Although the presence of another person can influence the latencies and the kinematic profiles of reach-to-grasp trajectories, specifically when intending to communicate or cooperate with the partner, it is not clear yet whether the social context modulates only those actions that are relevant in the current social situation (reaching, manipulating, and displacing objects) or whether the social context modulates all actions that are performed even when they are irrelevant according to the current social and communicative situation.

In the present study, we questioned the specific effect of social intention on movement kinematics for the main manipulative action but also for the preparatory action that was included in the procedure, to initiate each experimental trial. As such, we will be able to discuss whether the social intention induces a general state upon the social behavior or whether social intention has a more specific effect on the action that is carried out toward the target object. To test this hypothesis, participants were asked to reach and grasp as fast as possible an object and to move it to another location. Before performing this main action, participants were required to position the object on an initial pad. In contrast with the main action, this preparatory action was performed without any temporal constraint or direct social interaction. The effects of social context on the kinematic parameters of both the main and the preparatory actions were analyzed both when the actions were performed in absence and in the presence of another person, who could intervene on the target object or not depending on his relative position around the workspace, and on task instructions.

## MATERIALS AND METHODS

### PARTICIPANTS

Twenty-one healthy adults took part in the experiment (mean age = 22.7, SD = 4.8). All participants were right-handed, with a mean laterality coefficient of 0.88 (Edinburgh Handedness Inventory; Oldfield, 1971) and had no prior knowledge about the scientific aim of the study. Participants provided informed consent before participating in the experiment. The experimenter, a 23-year-old man, played the role of the social partner in all the social conditions requiring a second participant. The protocol followed the general ethics rules defined by the Helsinki guidelines for human experiments and was approved by the local institutional ethic committee.

### APPARATUS AND STIMULI

**FIGURE 1:**
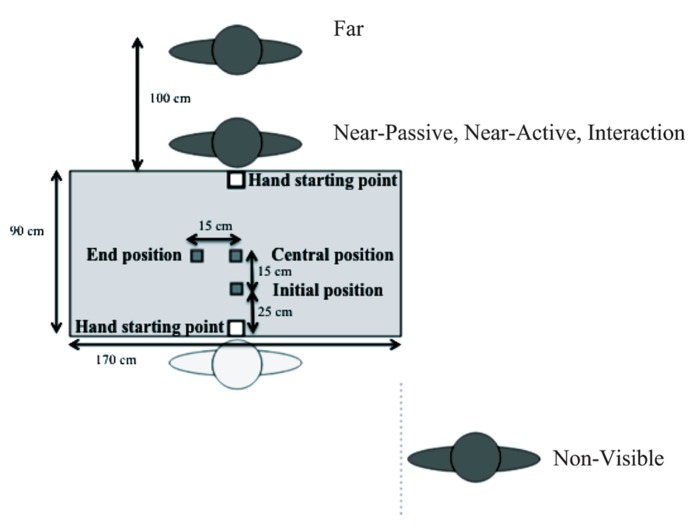
**Experimental setup showing the “initial,” “central,” and “end” positions as well as the respective distances.** The position of the participant (light gray) and the partner (dark gray) within the different experimental conditions (absent, near, far) are illustrated. The white squares indicate the starting hand positions for both the participant (*bottom*) and the experimenter (*top*).

Participants sat in front of a table (180 cm × 90 cm) on which red landmarks (3 cm × 3 cm) symbolized three specific locations that will be referred to in the next section as the initial position, the central position, and the end position (see **Figure 1**). In addition, two target-locations were placed on either side of the table, and were used to indicate the starting hand position for both the participant and the experimenter. The object that was to be manipulated was a wooden dowel (width 2 cm and height 4 cm), which was placed on the initial position at the beginning of each trial. In order to prevent any influence of verbal instruction, all trials were triggered through the emission of auditory tones broadcasted by computer speakers.

### PROCEDURE

During the experimental session, both the participant and the experimenter were seated on either side of the table, facing each other. The participants’ task was to reach and grasp the dowel between the thumb and the index finger in order to move it from one position to the other. Each trial started with the object placed at the initial position and with participants pinching their index finger and thumb together, with the fingertips set upon the starting hand position. A trial was defined as a series of three successive action sequences: *Preparatory Action*, which consisted in displacing the dowel from the initial to the central position (no temporal constraints), the *Main Action* which consisted in displacing as fast as possible the dowel from the central to the end position, and the *Repositioning Action* which consisted in displacing the dowel from the central to the initial position (no temporal constraints), making the setup ready for the next trial. Time pressure was set on the *Main Action* only and for this movement, the speed of the participants’ wrist was required to be superior to 80% of maximal speed (previously registered, see below). Each move was triggered by a different broadcast tone, which was always played in the same order (tone 1 initiated the *Preparatory Action*; tone 2 initiated the *Main Action*; tone 3 initiated the *Repositioning Action*). In order to prevent participants from anticipating the time of movement initiation, the time intervals between tones were randomized and lasted unpredictably between 1 and 3 s.

Tone 2, which initiated the *Main Action*, could be one of two pitches (low or high). When tone 2 was high-pitched, participants were to perform the *Main Action* as quickly and as accurately as possible. When it was a low-pitch tone, participants were to required to refrain from moving and the experimenter was to pick the dowel up from the central position and to place it on the end position as quickly and as accurately as possible.

### PRACTICE SESSIONS

All participants underwent two practice blocks before the experimental session started. A first practice block was performed to obtain an estimation of the maximum speed at which each participant could grasp the wooden dowel from the central position and place it on the end position. We used an adjustment procedure, which consisted in modifying the threshold (maximum speed) according to each participant’s performance level. If they were faster than the threshold computed on the last trial, the threshold was increased and reciprocally, if they were slower, it was decreased (by 50 mm.s^-^^1^ at the beginning of the adaptation phase and then, progressively by a smaller change until reaching a 5 mm.s^-^^1^ modulation, at the end of the adaptation phase). The practice block ended when the threshold did not increase or decrease more than three times during the five last consecutive trials, indicating that the threshold was near to the participants’ maximum speed. The mean value of the six last measurements was then taken as the individual’s speed reference for the *Main Action* in the experimental session. A second practice block (16 trials) was performed in interaction with the experimenter in order to assess whether the instructions were understood by the participants, that the different tones where clearly identified and that the appropriate motor responses were provided.

### EXPERIMENTAL CONDITIONS

In order to test the contrasting effects of the four different social contexts that were targeted in this study, we designed five experimental conditions in which the experimenter was placed in different places around the table with respect to the participant (see **Figure 1**). Participants took part in all five conditions following a randomized block design.

#### Absent

The experimenter was not visible while participants performed the pick and place task. Tone 2 was always a high-pitch sound and thus, all *Main Actions* were performed by the participant.

#### Far

The experimenter was seated on a chair, facing the participants, at a distance of 100 cm from the table. At the start of the block, the experimenter stretched out his right arm to show the participants that he could not reach the table center. Tone 2 was always a high-pitch sound and thus, all *Main Actions* were performed by the participant.

#### Near-passive

The experimenter was seated at the table, facing the participant. At the start of the block, the experimenter stretched out his right limb to show the participants that he could reach the table center, though he stayed totally immobile throughout the entire experimental session. Tone 2 was always a high-pitch sound and all *Main Actions* were performed by the participant.

#### Near-active

The experimenter was seated at the table, facing the participant. At the start of the block, the experimenter stretched out his right arm to show the participants that he could reach the table center. Tone 2 was always a low-pitch sound and thus, all *Main Actions* were performed by the experimenter.

#### Interaction

The experimenter was seated at the table, facing the participant. At the start of the block, the experimenter stretched out his right limb to show the participants that he could reach the table center. Tone 2 was a high-pitch sound in 70% of the *Action* trials and was a low-pitch sound in the remaining 30%. Thus, the *Main Actions* were performed by the participant in 70% of the trials.

A given condition ended when a score of 20 points was achieved. Each point was obtained when a correct *Main Action* was performed, i.e., when the motor performance satisfied the temporal, spatial and social constraints.

### DATA RECORDING AND ANALYSIS

The participants’ movements were recorded using four Oqus infrared cameras (Qualisys System). Kinematics of reach-to-grasp and transport movements were measured by recording the 3D displacement of the five infrared reflective markers that were placed on the index (base and tip), the thumb (tip), and the wrist (scaphoid and pisiform) of the participant. One additional marker was placed on the dowel. Cameras were calibrated before each session, allowing the system to reach standard deviation accuracies smaller than 0.2 mm, at a 200-Hz sampling rate.

**FIGURE 2:**
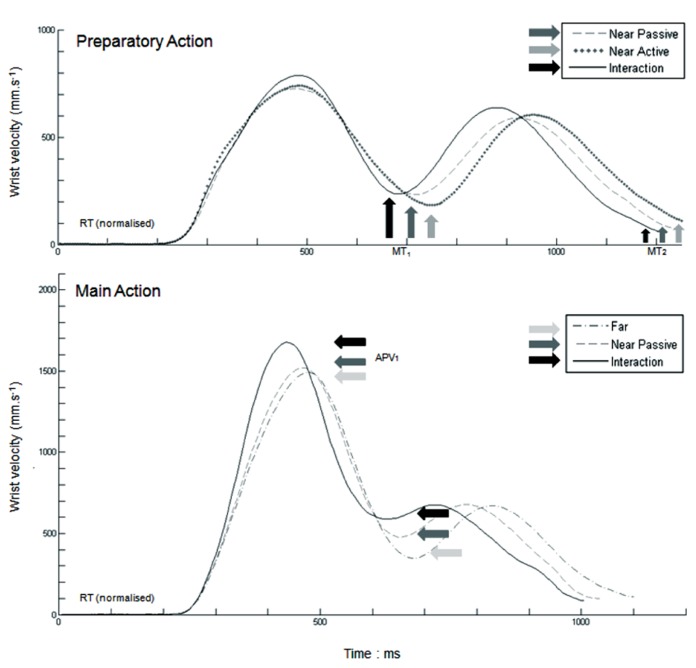
**Mean kinematic patterns for a typical participant in the different experimental conditions.** All patterns are synchronized to the initiation time. On the* preparatory action* (top), we observed both an effect of social uncertainty (Near-passive and Near-active vs Interaction) and an effect of social intention (Near-passive vs Near-active) on the first movement time. On the *Main Action* (bottom), results showed that accelerative part of the second movement is modified when acting in the partner reachable space (Far vs Near-passive and Interaction). Moreover, social uncertainty (Near-passive vs Interaction) affect strongly the first movement time and the first peak of velocity.

From these measures, tangential 3D instantaneous velocity profiles were calculated. All movements (*Preparatory Action*, *Main Action*, *Repositioning Action*) were characterized by two bell-shaped profiles (see **Figure 2**). The first bell-shape curve corresponded systematically to the movement of reaching to pick the target object, which will be referred to in the following as the first movement of the sequence. The second bell-shape curve corresponded to the movement of lifting to place the target-object, which will be referred to in the following as the second movement of the sequence. For both movements, kinematic parameters of the arm and of the grip components were measured. As classically used in previous studies, reaction time (RT), trajectory amplitude, and early kinematic parameters (amplitude and time to peak of acceleration and velocity phases) were here used because they inform on the motor planning properties, whereas movement time and trajectory smoothness (as revealed by jerk analysis) inform on the guiding strategies that are used to displace the hand through action space. These parameters have been pointed out to be relevant indicators for human observers that were required to extract meaningful interaction-cues when viewing point-light displays (e.g., Pollick et al., 2001; Cook et al., 2009). Definitions and codings of the selected parameters are presented in **Table 1**.

**Table 1:** Definition of the different kinematic parameters considered in the study.

Name	Code	Unit	Definition
Reaction time	RT	ms	Time duration between tone onset and first moment in time for which velocity of wrist marker was greater than threshold of 20 mm.s^-^^1^
Movement time of Mvt_1_	MT_1_	ms	Time duration of the first element of the sequence, equals to the moment in time for which the local minima between the two “bells” occurs minus the reaction time
Amplitude of peak velocity of Mvt_1_	APV_1_	mm.s^-^^1^	Amplitude of the first peak of velocity (first zero crossing of acceleration)
Time to peak velocity	TPV_1_	ms	Moment in time for which the first peak of velocity occurs minus the reaction time
Amplitude of peak acceleration of Mvt_1_	APA_1_	mm.s^-^^2^	Amplitude of the maxima of the first derivate of velocity between the start of movement and the peak of velocity
Mean jerk during acceleration phase of Mvt_1_	Jerk_1_	mm.s^-^^3^	Mean of absolute values of jerk: second derivate of velocity between the start of the movement and the peak of velocity
Amplitude of peak height of Mvt_1_	APH_1_	mm	Amplitude of the first maximum value from *Z*-axis data during element 1
Maximum grip aperture	MGA	mm	Amplitude of the maximum of the distance between index and thumb marker during element 1
Time to maximum grip aperture	TGA	ms	Moment in time for which maximum grip aperture occurs

**MT*_2_, APV_2_, TPV_2_, APA_2_, Jerk_2_, and APH_2_ are the same kinematic parameters as above but extracted from Mvt_2_ (second bell-shape on velocity profiles for Action trials).*

In the present study, we report the analyses that were conducted on the *Preparatory Action* and *Main Action* only. The *Repositioning Action* was not analyzed. For each participant and condition, the kinematic parameters were submitted to a repeated-measure ANOVA with the five-level Condition as within factor. The alpha level of significance was set to 0.05. To further investigate the main effect of Condition, we used a posteriori contrasts (see details of matrix coefficients in **Table 2**). More specifically, we tested the *effect of audience* by opposing *Absent* against all other conditions (Ψ_1_). We operationalized the *effect of sharing reachable space* by opposing *Far* against those conditions for which the experimenter was sitting at the table (Ψ_2_). We tested the *effect of social uncertainty* by opposing *Interaction* against the conditions for which there was no ambiguity about who was required to perform the *Main Action* (Ψ_3_). Finally, for the *Preparatory Action*, we tested the *effect of social intention* by opposing *Near-passive* and *Near-active* conditions (Ψ_4_). As these four contrasts are orthogonal, they are independent and will provide the means to assess the explanatory power of each contrast for a given main effect.

**Table 2:** Presentation of the orthogonal *post hoc* contrasts that were used to assess the social effects in the *Preparatory Action* and the *Main Action*, respectively.

Contrast	Non-visible	Far	Near-passive	Near-active	Interaction	ΣCa
***Preparatory Action***
Ψ_1_ audience	+4	-1	-1	-1	-1	0
Ψ_2_ space	0	+3	-1	-1	-1	0
Ψ_3_ uncertainty	0	0	-1	-1	+2	0
Ψ_4_ intention	0	0	+1	-1	0	0
***Main Action***
Ψ_1_ audience	+3	-1	-1	0	-1	0
Ψ_2_ space	0	+2	-1	0	-1	0
Ψ_3_ uncertainty	0	0	+1	0	-1	0

## RESULTS

### PREPARATORY ACTION

Concerning the *Preparatory Action* we observed a global effect of Condition on RT [*F*(4,80) = 21.458, *p* < 0.001, ŋ_p_^2^ =0.52] and TGA [*F*(4,80) = 6.548, *p* = 0.019, ŋ_p_^2^ = 0.14]. For the first movement of the sequence, the effects of Condition was also significant on MT_1_ [*F*(4,80) = 3.257, *p* = 0.016, ŋ_p_^2^ = 0.14], TPV_1_ [*F*(4,80) = 3.103, *p* = 0.020, ŋ_p_^2^ = 0.13], Jerk_1_ [*F*(4,80) = 2.579, *p* = 0.044, ŋ_p_^2^ = 0.11], APH_1_ [*F*(4,80) = 3.317, *p* = 0.014, ŋ_p_^2^ = 0.14]. For the second movement of the sequence, the effect of Condition was significant on APH_2_ [*F*(4,80) = 3.450, *p* = 0.012, ŋ_p_^2^ = 0.15]. No effects were found on end-point errors [*F*(4,80) = 1.41, *p* = 0.236], indicating that the end-point accuracy was maintained constant throughout all experimental conditions and thus, did not provide any account for the effects observed on motor kinematics. These results indicate that the presence, the location and/or the interaction with the experimenter were taken into account during motor planning and modulated motor execution. The mean values of the different kinematic parameters are presented in **Table 3**. To obtain more specifics about the effects that were impacting movement parameters, we conducted a series of *post hoc* contrast analyses.

**Table 3:** Mean values for the different kinematic parameters (with standard error in parentheses).

	Preparatory Action						Main Action			
	Absent	Far	Near-passive	Near-active	Interaction		Absent	Far	Near-Active	Interaction
RT	334 (15)	328 (13)	320 (10)	410 (12)	360 (12)		210 (6)	220 (8)	226 (6)	267 (9)
						*Grasping*			
TGA	331 (10)	326 (8)	331 (9)	341 (8)	325 (10)		344 (13)	350 (14)	346 (14)	329 (14)
MGA	80.7 (1.7)	80.5 (2.1)	80.9 (2.1)	81.2 (1.9)	79.4 (1.6)		94.4 (3.3)	91.8 (3.1)	92.7 (3.1)	92.9 (3.2)
						*Reaching*				
APV_1_	710 (22)	717 (19)	711 (23)	708 (19)	721 (27)		1503 (56)	1498 (50)	1504 (54)	1579 (61)
TPV_1_	227 (7)	229 (6)	229 (5)	237 (6)	226 (7)		235 (8)	236 (8)	236 (8)	223 (9)
MT_1_	423 (13)	421 (11)	422 (12)	438 (12)	417 (11)		447 (12)	450 (14)	445 (14)	431 (15)
APA_1_	5608 (305)	5480 (342)	5662 (321)	5637 (296)	5970 (386)		11171 (853)	10836 (743)	11207 (879)	12511 (1032)
Jerk_1_	3206 (178)	3169 (155)	3159 (158)	3042 (152)	3322 (203)		6507 (453)	6382 (396)	6497 (450)	7235 (517)
APH_1_	61.5 (1.6)	59.8 (1.6)	60.4 (1.7)	62.8 (1.5)	61.1 (1.7)		68.4 (2.1)	67.1 (2.0)	67.1 (2.1)	68.0 (2.1)
						*Placing*				
APV_2_	719 (15)	725 (14)	715 (16)	702 (17)	715 (16)		765 (22)	770 (24)	757 (24)	751 (23)
TPV_2_	171 (6)	174 (5)	173 (5)	173 (6)	173 (6)		127 (6)	130 (6)	126 (5)	125 (6)
MT_2_	499 (14)	505 (16)	498 (15)	517 (16)	509 (18)		365 (11)	363 (10)	364 (10)	357 (11)
APA_2_	4132 (229)	4169 (198)	4086 (210)	4059 (184)	4144 (209)		4391 (317)	4507 (282)	4165 (266)	4016 (266)
Jerk_2_	2343 (117)	2373 (106)	2282 (106)	2293 (98)	2357 (114)		2597 (188)	2626 (166)	2442 (163)	2339 (166)
APH_2_	67.7 (2.2)	66.6 (2.2)	67.4 (2.1)	72.6 (2.5)	68.3 (2.6)		74.3 (2.6)	70.9 (1.7)	72.4 (2.1)	73.4 (2.2)

*Results are presented for each experimental condition for the *Preparatory Action* and for the *Main Action*, respectively.*

#### Effect of audience

No kinematic parameters were found to be significantly affected when comparing the Absent condition vs the three other conditions. RT was found to be only close to significance (*t* = 1.947, *p* = 0.065) thus suggesting no audience effects on RT. In agreement with this, we observed an absence of Condition effect on all 16 kinematic parameters, confirming a weak audience effect on motor performances.

#### Effect of sharing reachable space

The results showed an effect of reachable space on RT when contrasting the conditions (Far) and (Near-passive, Near-active, and Interaction). Participants performed the *Preparatory Action* with a longer RT (*t* = 3.78, *p* = 0.001) in the Far condition. We also found that the increase in RT in all the Near conditions was the most significant for the Near-active (*M* = 410 ms, SD = 55 ms) and Interaction conditions (*M* = 360 ms, SD = 55 ms) as compared to the Far condition (*M* = 328 ms, SD = 60 ms). No differences were found between Far and Near-passive conditions (*M* = 320 ms, SD = 46 ms, *p* = 0.979), suggesting that the observed effects were supported by other more specific and independent variables (e.g., social interaction). No effects on MT or kinematic parameters were observed. Thus, we hypothesized that the global effect on kinematics reported above were not due to the near presence of the partner but rather due to the interactive process that takes place during the other experimental conditions. To verify this hypothesis, we dissociated two contrasting hypotheses within the three “Near” conditions. First, we tested the effect of social uncertainty by contrasting (Interaction) vs (Near-passive and Near-active) conditions considered together. Second, we tested the effect of social intention by contrasting the conditions (Near-passive) vs (Near-active).

#### Effect of social uncertainty

When contrasting (Interaction) vs (Near-passive and Near-active) conditions, the results showed an effect of social uncertainty on the kinematic parameters of the first movement of the sequence with shorter MT_1_ (*t* = 2.756, *p* = 0.012), shorter TPV_1_ (*t* = 3.611, *p* = 0.002), higher Jerk_1_ (*t* = 2.735, *p* = 0.128), and shorter TGA (*t* = 2.427, *p* = 0.025) in the Interaction condition compared to the two other conditions considered together. Because all aspects of the task were maintained identical (i.e., starting position, relative positions of participant and experimenter, object location and size, end-position and end-point accuracy) but the social context, the only variable that could account for these results was the uncertainty of whether the next movement would be performed by the participant or by the experimenter. Moreover, in the *Preparatory Action* condition the audio stimulus was strictly the same regardless of the condition (near-active, near-passive, and interaction). Thus, the effects reported could not be accounted for by a stimulus–response contingency effect but would be more related to the social situation *per se*.

#### Effect of social intention

When participants initiated the task under the Near-passive condition, results revealed a significant shorter RT (*t* = 10.823, *p* < 0.001) and shorter TGA (*t* = 2.727, *p* = 0.013) than when participants initiated the task under the Near-active condition. For the first movement, a shorter MT_1_ (*t* = 2.918, *p* = 0.009), a lower APH_1_ (*t* = 2.424, *p* = 0.025) was also observed along with a lower APH_2_ (*t* = 2.510, *p* = 0.021) for the second movement in the Near-passive compared to the Near-active conditions. These results indicate that even though the “motor” intention is the same, the “social” intention involved in the task is taken into account during the planning of the *Preparatory Action*, as reflected in the kinematic parameters of both the first and the second components of the action sequence.

### MAIN ACTION

When considering the *Main Action*, the statistical analyses revealed a global effect of Condition on RT [*F*(3,60) = 33.806, *p* < 0.001, ŋ_p_^2^ = 0.63] and TGA [*F*(3,60) = 6.548, *p* < 0.001, ŋ_p_^2^ = 0.25] as well as on five other kinematic parameters characterizing the first movement of the sequence, i.e., APV_1_ [*F*(3,60) = 7.814, *p* < 0.001, ŋ_p_^2^ = 0.28], TPV_1_ [*F*(3,60) = 8.690, *p* < 0.001, ŋ_p_^2^ = 0.30], MT_1_ [*F*(3,60) = 3.827, *p* = 0.014, ŋ_p_^2^ = 0.16], APA_1_ [*F*(3,60) = 9.076, *p* < 0.001, ŋ_p_^2^ = 0.31], and Jerk_1_ [*F*(3,60) = 11.397, *p* < 0.001, ŋ_p_^2^ = 0.36]. For the second movement of the sequence, results revealed an effect of Condition on APA_2_ [*F*(3,60) = 3.326, *p* = 0.026, ŋ_p_^2^ = 0.14] and Jerk_2_ [*F*(3,60) = 3.816, *p* = 0.014, ŋ_p_^2^ = 0.16] only. No effects of Condition were revealed on any of the other kinematic parameters, MGA or end-point errors.

Because all aspects of the task were maintained identical throughout all conditions (i.e., starting position, relative positions of participant and experimenter, object location and size, end-position and end-point accuracy) except for the social context, these findings strongly suggest a global planning of the motor sequences during which the social context is taken into account, with as a consequence the modulation of the kinematic properties of both movements of the action sequence. To gather more information about the specific effects and the role played by the social context on these effects, we conducted a series of *post hoc* contrast analyses according to the three hypotheses mentioned above.

#### Effect of audience

When comparing Absent vs the three other conditions, we found an effect of audience on RT (*t* = 6.01, *p* < 0.001). Participants initiated movements faster in the Absent condition (*M* = 210 ms, SD = 25 ms) compared to the Far (*M* = 220 ms, SD = 35 ms), Near-passive (*M* = 226 ms, SD = 27 ms), and Interaction (*M* = 267 ms, SD = 42 ms) conditions. The audience effect did not have a significant effect on any other of the kinematic parameters. Overall these findings suggest that, as for the *Preparatory Action*, when taken independently from the other effects (space, uncertainty), the mere presence of a partner had little effect on motor kinematics.

#### Effect of sharing reachable space

When contrasting the conditions (Far) vs (Near-passive and Interaction), statistical analyses revealed that APA_2_ (*t* = 2.48, *p* = 0.022) and Jerk_2_ (*t* = 2.40, *p* = 0.026) were greater when the partner was far from the participants than when he was near (APA_2_: *M* = 4507 mm.s^-^^2^, SD = 1290 mm.s^-^^2^; Jerk_2_: *M* = 2626 mm.s^-^^3^, SD = 760 mm.s^-^^3^). Indeed, both Near-passive (APA_2_: *M* = 4165 mm.s^-^^2^, SD = 1220 mm.s^-^^2^; Jerk_2_: *M* = 2442 mm.s^-^^3^, SD = 748 mm.s^-^^3^) and Interaction conditions (APA2: *M* = 4016 mm.s^-^^2^, SD = 1220 mm.s^-^^2^; Jerk_2_: *M* = 2339 mm.s^-^^3^, SD = 761 mm.s^-^^3^) showed small APA_2_ and low Jerk_2_, indicating a more fluent transport phase during the sequential action when performed within the partner’s reachable space.

#### Effect of social uncertainty

When contrasting the conditions (Interaction) vs (Near-passive), participants were characterized by longer RT (*M* = 267 ms, SD = 42 ms vs *M* = 226 ms, SD = 27 ms, *t* = 5.44, *p* < 0.001) and shorter TGA (*M* = 329 ms, SD = 65 ms vs *M* = 346 ms, SD = 65 ms, *t* = -4.96, *p* < 0.001). Data analyses also revealed higher APV_1_ (*M* = 1578 mm.s^-^^1^, SD = 280 mm.s^-^^1^ vs *M* = 1504 mm.s^-^^1^, SD = 246 mm.s^-^^1^, *t* = 4.13, *p* < 0.001), shorter MT_1_ (*M* = 431 ms, SD = 67 ms vs *M* = 445 ms, SD = 63 ms, *t* = 3.39, *p* = 0.003), shorter TPV_1_ (*M* = 223 ms, SD = 40 ms vs *M* = 236 ms, SD = 38 ms, *t* = 5.11, *p* < 0.001), higher APA_1_ (*M* = 12511 mm.s^-^^2^, SD = 4728 mm.s^-^^2^ vs *M* = 11207 mm.s^-^^2^, SD = 4028 mm.s^-^^2^, *t* = 4.53, *p* < 0.001), and higher Jerk_1_ (*M* = 7235 mm.s^-^^3^, SD = 2367 mm.s^-^^3^ vs *M* = 6497 mm.s^-^^3^, SD = 2064 mm.s^-^^3^, *t* = 5.08, *p* < 0.001) in the Interaction condition compared to that observed in the Near-passive condition. Furthermore, data analysis testing for the effects of Condition on MT_2_ was close to significant (*t* = 2.00, *p* = 0.059) with a tendency for shorter MT_2_ (*M* = 357 ms, SD = 50 ms vs *M* = 365 ms, SD = 48 ms) in the Interaction condition compared to that measured in the Near-passive condition. These results suggest a global effect of social uncertainty with longer RTs, and faster and less fluent action execution when acting under the uncertainty that the partner may perform the main action (in 30% of trials). However, these effects were mainly observed on the first movement with little effects on the second.

## DISCUSSION

The aim of the present study was to evaluate the influence of reachable space, social uncertainty and social intention on movement kinematics characterizing a sequential manipulative action that consisted in placing a dowel (preparatory action) before performing a temporally constrained task (main action) that required participants to move as fast as possible the dowel from one location to another. The analyses of the kinematic patterns of both the preparatory (executed under no constraints) and the main action (executed under speeded constraints) revealed an absence of influence of the mere presence of a partner, i.e., the audience effect was negligible. However, there was a significant effect of the social context with variations of movement kinematics of the main action but also of the preparatory action when the partner was located close enough to the table to be able to intervene on the object. Overall, our data suggest a specific effect of the social risk of “sharing reachable space.” In the following sections, we will quickly review the reported results and propose a discussion on the importance of these findings for the field of neuro-robotics.

Using a rather simple reach-to-grasp task, we manipulated the effect of audience, the effect of sharing reachable space, the effect of social uncertainty and the effect of social intention. First, although it is well established that the mere presence of a partner can affect participants’ behavior (Zajonc, 1965), results showed that the presence of a potential partner was not sufficient to affect the kinematics of the grasping and placing phase of a manipulative task (*Main Action*). These results are in agreement with earlier studies, which reported that movement kinematics is affected by the presence of another person only when an interaction between the two agents can occur (Georgiou et al., 2007; Becchio et al., 2008a,b). In contrast, we observed that the presence of a partner sharing the participants’ reachable space had a significant effect on the properties of movement kinematics with longer RTs and lower acceleration peaks, which rendered the arm trajectories less jerky (more fluent). These findings suggest that the presence of a partner sharing reachable space lead the actor to slow down the motor planning process in order to enhance movement guiding strategies, resulting thus in a more fluent transport phase of the sequential action. To note is the fact that these patterns of results were observed essentially for the second element of the main action (i.e., the transport phase). At first, it may be thought that these results suggest that kinematic modulations were associated to the space variability of the object that is placed on the table. However, through the use of real-time control for small error acceptance, we controlled for this factor: the kinematic variations could not be due to the end-point accuracy constraints and may in fact directly be related to the experimental conditions. In agreement with previous work (Gianelli et al., 2013), these findings indicate that grasping an object to transport it to a new location is affected by whether this object is located in someone else’s reachable space, notwithstanding the fact that the aim to interact is made explicit or not. Hence, the fact that movements were smoother and performed with lower acceleration profiles when executed in other’s reachable space suggests that grasping actions are influenced by the possibility of experiencing a social interaction.

The main finding of the present study is, however, the fact that social context influenced not only the kinematics of the main action but also the kinematics of the preparatory action for which no instructions were given for temporal, spatial, or social constraints and despite the fact that this movement was entirely performed out of the reachable space of the partner. Overall, we report in the present study similar effects of social uncertainty in both the *Preparatory Action* and the *Main Action*, showing that the interaction condition not only influenced the grasping task performed as fast as possible (in order to be rewarded by points), but also the preparatory sequence of this action, which was performed always by the participant. Social uncertainty led participants to perform the preparatory actions faster, resulting in earlier time to peak velocity and grasp aperture as well as increased jerk. These results indicate that participants felt an urge to perform the preparatory action with shorter response times when the experimental condition generated ambiguity about who will then act. Indeed, in the interaction condition, during the preparatory actions, participants did not know who was going to perform the main action since the sound indicating the agent was given after the preparatory action had been executed. Hence, social uncertainty led participants to adopt a general competitive behavior, which has been previously described in paradigms that are, however, usually designed specifically to encourage direct competition (Georgiou et al., 2007). Effect of the social context on movement kinematics was also observed during actions for which the object was placed in totally predictive contexts: data showed that participants tended to have longer RTs and movement times, and performed more curved trajectories (e.g., higher wrist displacement, APH) when they positioned the object for a forthcoming social action performed by a partner in 100% of the trials (Near-passive condition) rather than individually (Near-active condition). Variations in movement kinematics are then observed when participants place the object knowing that the partner is going to grasp it and when they place the object knowing that they will personally have to grasp it. Slower actions and higher wrist trajectories may have been implemented to attract the partner’s attention and give the person time to prepare the interactive response (Sartori et al., 2009a). Interestingly, this pattern of results was obtained even if the motor intention was identical throughout, i.e., an identical target and a similar motor task. It confirms the influence of social intention on movement kinematics, as already reported by Becchio et al. (2008a), and further demonstrates the effect of social intention on motor behaviors as a global effect that affects both the early and the late portion of the execution of the motor sequence.

These results reinforce the importance in computational motor control to take into account the contextual constraints such as reachable space, environment predictability, and social intentions. Current models of motor control (for a review, see Todorov, 2004) are based on optimized function costs that are often named minimum-X (jerk, torque change, energy, time, variance, etc.). The present results further demonstrate that such optimized function cannot account for the specific effects that we have reported both for the main and the preparatory actions. Here, we confirm that interacting with a partner encompass different processes that may be independent from each other. First, the effect of reachable space was found to be a “localized” effect on kinematics only revealed when the movement was directly made within the reachable space of the conspecific. The observed consequence is that the accelerations (APA_2_ – Jerk_2_) were reduced giving rise to smoother movements. This could be a consequence of years of learning that when acting within the reachable space of someone else, the agent must have smoother movements in order to not frighten the partner away, smoother and slower profiles being perceived as more gentle and socially engaging actions. This specific learning could be shaped during the early developmental years when young children are interacting with their parents, individuals who are there to teach how to “be gentle” during social interactions (Gaussier et al., 1998; Hasnain et al., 2012). Second, the effect of social uncertainty is found to be a “global” effect on kinematics, neither localized to a specific part of the sequence, nor to a specific spatial location between the participant and the partner. We found that when the agent cannot entirely predict who will perform the next Main Action, (s)he was performing voluntary actions as if they were in a competitive interaction and thus, modulated both the first and the second components of the motor sequence resulting in less smooth movements (higher accelerations; higher jerks). More experiments are now needed to better understand how the perception of a competitive situation in relation to the social context may influence the kinematics of voluntary motor actions. Third, we revealed an effect of social intentions independently from the previous effects. In our case, the preparatory action showed specific patterns of movement curvature with higher wrist displacements and slower movements when participants placed the object to be grasped by the partner compared to the situation for which the object was placed for self-use. Because this situation led to a less “optimized” motor performance, one may speculate that this strategy would be employed as an external signal during social interaction to show the agent’s social intention to share the object (Sartori et al., 2009a). Previous studies have supported this interpretation by showing that humans are sensitive to external kinematic characteristics of a movement, and especially trajectory height (Manera et al., 2011; Sartori et al., 2011; Lewkowicz et al., 2013). The new findings reported here demonstrate that even preparatory actions reflect the agent’s social intention and thus, movement properties may be *read* by perceivers for whom understanding motor intention from early kinematics is important. This is at least one of the key elements lacking today in humanoid robot systems because they are not implemented at the moment with the appropriate embedded perceptual system that can take advantage of these early motor cues.

In conclusion, the present study provides the first report of a social effect on kinematics in a non-constraint action. To summarize, we found that the mere presence of a conspecific did not influence the preparatory action, even when sharing reachable space with that of the actor, but an overall effect was observed when the task involved social uncertainty and social intention. This result is important as it shows that social uncertainty and intentionality influence kinematics very early on during motor planning, and may thus represent a highly informative signal in the case of cooperative and competitive social situations (see also Manera et al., 2011). These empirical results can have significant impact in the field of neuro-robotics as they suggest that acting in a social interactive environment leads to a certain number of parameters that impact movement kinematics directly: reachable space, uncertainty, and social intention. These effects may constitute what humans perceive as a “social interactive” situation, effects that need to be taken into account to create robots with what is called today as intuitive interactivity. Future studies need now to consider how to implement these social aspects of motor control within an artificial system in order to afford intention reading during human–robot collaborative work. More specifically, the questions of low/high-level kinematics and explicit/implicit learning will be the key to implement intuitivity in future humanoid robots.

## Conflict of Interest Statement

The authors declare that the research was conducted in the absence of any commercial or financial relationships that could be construed as a potential conflict of interest.
